# Novel Hybrid Biomass Anti-Aging Filler for Styrene-Butadiene Rubber Composites with Antioxidative and Reinforcing Properties

**DOI:** 10.3390/ma13184045

**Published:** 2020-09-11

**Authors:** Xiaohui Guo, Yuanfang Luo, Yongjun Chen, Lijuan Chen, Demin Jia

**Affiliations:** 1 Key Lab of Guangdong High Property and Functional Macromolecular Materials, Department of Polymer Materials and Engineering, South China University of Technology, Guangzhou 510640, China; msgxh@mail.scut.edu.cn (X.G.); yjchen@scut.edu.cn (Y.C.); psdmjia@scut.edu.cn (D.J.); 2 Center for Advanced Analytical Science, c/o School of Chemistry and Chemical Engineering, Guangzhou University, Guangzhou 510006, China; 3 School of Chemistry and Chemical Engineering, Qiannan Normal University for Nationalities, Duyun 558000, China

**Keywords:** rubber composites, anti-aging filler, silica, biomass, tea polyphenol, thermostable

## Abstract

Antioxidants are normally utilized to extend the service life of polymers due to the strong reducibility of the phenolic hydroxyl group of the hindered phenol structure. Inspired by this characteristic, we have introduced green tea polyphenol (TP) supported on a silica surface containing considerable phenolic hydroxyl groups to obtain a novel biomass anti-aging filler (BAF, denoted as silica-s-TP) to reinforce and improve the anti-aging property of rubber composites. The applying of silica-s-TP to enhance the thermal-oxidative stability and ultraviolet light (UV) aging resistance of styrene-butadiene rubber (SBR) was evaluated. The hybrid biomass anti-aging filler could not only uniformly disperse in the rubber matrix, giving rise to the excellent mechanical properties, but also enhance the properties of thermal-oxidative stability and UV aging resistance with the increasing silica-s-TP content of SBR distinctly. This study provides a mild and environmentally friendly strategy to prepare the functional biomass filler, which could be applied as not only a reinforcement filler but also an anti-aging additive in “green rubber”.

## 1. Introduction

Polymeric materials aging is a crucial problem for its long period applications. Polymer aging is caused by heat, especially under high temperatures for a long time, an excess of oxygen, chemicals, and ultraviolet (UV) radiation [1]. The accompanying variation worsens the properties and stability of polymeric materials and restrict their applications to a great extent. Namely, aging of polymeric materials tends to accelerate the destruction of the material’s properties, leading to a reduction in the service life and an increase in the consumption of resources and, under certain circumstances, can be catastrophic. A particularly obvious example is the aging of rubber tires. Diene elastomers, such as natural rubber (NR), butadiene rubber (BR), and styrene-butadiene rubber, are important elastomers in modern industry [2,3]. The main chain of rubber contains unsaturated chains and allyl hydrogen, which are prone to thermal oxidative aging and molecular chain break [4,5]; oxidative aging is the most common [6,7]. For preventing oxidative aging of rubber material and prolonging its service life, anti-aging agents have been applied to inhibit and eliminate the free radicals. However, some commercial anti-aging agents can play a role to a certain extent, but there are some shortcomings that limit its application, such as poor antioxidant efficiency, volatility, and easy migration. Moreover, most oxidants are toxic, and will cause certain harm to people and the environment [8,9]. Therefore, it is of certain research significance to seek non-toxic and natural anti-aging agents.

Amine and phenolic antioxidants are commonly used in rubber anti-aging systems [10,11,12,13]. Compared to the amine antioxidant, phenolic antioxidants are suitable for colorless or light-colored rubber products due to their non-polluting and non-discoloration characteristics. As is well known, polyphenolic compounds and phenolic are present in a large number of plants, including tea, coffee, vegetables, and unripe fruits. Tea polyphenols are the main biologically active ingredients in green tea and the main component of TPs is catechins. The catechins are mainly composed of (−)-epicatechin (EC), (−)-epicatechin gallate (ECG), (−)-epigallocatechin (EGC), and (−)-epigallocatechin gallate (EGCG). In addition, as a kind of biomass, TP is widely used as an antioxidant [13,14], UV protective agent, anticancer medicine [15], antibacterial drug [16,17,18], and reducer of graphene oxide due to its high reactivity of hydroxyl substitution and free radicals, and scavenging ability [19]. Yan et al. doped tea polyphenols into polyaniline molecular chains as a new type of efficient dopant and thermal stabilizer. Compared with pure polyaniline, doping TP into the molecular chain of polyaniline enhances the interactivity of the chain segments and promotes electron delocalization [20]. Guo et al. used tea polyphenol compounds to reduce graphene oxide to obtain the tea polyphenol reduced graphene (TPG). Using a direct slurry compounding method, the TPG slurry is uniformly dispersed in the chlorosulfonated polyethylene (CSM) to prepare a CSM/TPG composite. The study found that there is a strong interface interaction between CSM and TPG, which significantly improves the mechanical properties of the composite material [19,21]. Additionally, Guo et al. have used tea polyphenol compounds as reducing agents and stabilizers to functionalize graphene (JPTG), which is prepared by the Mannich reaction with graphene oxide. The nitrile rubber/JTPG composite is prepared by the acetone solution method, and the mechanical properties and electrical conductivity of the material are greatly improved [22].

Inorganic filler is a necessary ingredient for rubber products to strengthen the rubber matrix and cut down the cost. In past years, a large number of studies have indicated that silane coupling agent-modified inorganic filler could widely enhance the dispersion of inorganic filler in the rubber matrix [23]. Recently, a novel method of inorganic filler surface modification by the low molecular weight rubber additives on its surface has been established as an effective approach to obtain the combining performance of rigid filler and rubber additives [24]. For instance, the literature reports that inorganic filler surfaces modified by rubber antioxidants can realize homogeneous dispersion of the filler and improve the interface combination between the rubber and filler [25]. However, according to the relevant research, there are rare reports on tea polyphenol-functionalized silica. In addition, the effects of the tea polyphenol anchored on silica surface on the anti-aging and reinforcement properties of rubber have not been reported by researchers. Considering the reinforcing performance of silica, tea polyphenol biomass functionalized silica can provide better improvement of the final mechanical properties and antioxidant effects of rubber nanocomposites.

In this paper, a novel kind of TP-modified silica (silica-s-TP) as a biomass anti-aging filler, instead of conventional organic anti-aging additives, was introduced to the SBR matrix to simultaneously enhance the performance of thermo-oxidative aging and mechanical properties. The influences of biomass anti-aging filler on the dispersion, interfacial adhesion, mechanical properties, and anti-aging properties of SBR composites were systematically studied. As we expected, the silica-s-TP exhibited an excellent rubber reinforcement and anti-aging properties than the traditional amine or phenolic rubber anti-aging agents with the equal filler content due to the combined advantages of filler and biomass anti-aging agent through the chemical bonding between silica and TP. The aims of this work are to prepare a novel hybrid biomass filler that could be applied as a kind of non-toxic anti-aging additive with excellent antioxidative and reinforcing properties for the “green rubber” industry.

## 2. Experimental

### 2.1. Materials

SBR (1502) was produced from Guangzhou Institute of Rubber Products, Guangzhou, China. Tea polyphenol (TP) was obtained from Shenzhen Shenghai Bioengineering Co., Ltd., Shenzhen, China. Pristine silica (FINE-SIL 518) with the specific surface area of 200–220 m^2^/g was purchased from Huiming Chemical Co., Ltd., Jiangxi, China. Activator such as stearic acid (SA) and zinc oxide (ZnO), accelerator N-cyclohexylbenzothiazole-2-sulphenamide (CBS), and vulcanizator insoluble sulfur (S) were industrial grade products and used as received. Dibutyltin dilaurate (DBTDL), absolute ethanol were analytical reagents and used as received.

### 2.2. Preparation of an Organic-Inorganic Hybrid Biomass Anti-Aging Filler

**Figure 1:**
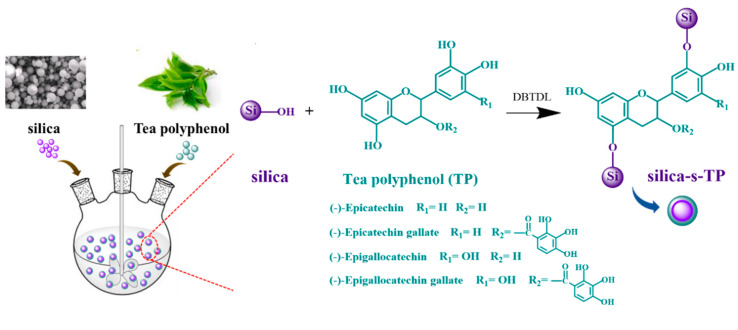
The synthesis route of silica-s-TP.

The synthesis route of biomass anti-aging filler (silica-s-TP) was shown in Figure 1. Silica-s-TP was prepared by a mild and one-step method. 15.0 g silica was added to 500 mL three-necked flask and dispersed in 300 mL absolute ethanol, and then 1 g TP and several drops of DBTDL were added into the suspension. After stirred at 50 °C for 11 h, the mixture was filtered and washed with ethanol for 4 times. Then, the product was dried in vacuum oven at 80 °C to constant weight.

### 2.3. Preparation of SBR/Silica-s-TP Composites

SBR composites prepared by filling different content of silica and silica-s-TP fillers were mixed with activator, accelerator and vulcanizator at room temperature for 10 min by a two-roll mill, respectively. The components of SBR/silica-s-TP composites are listed in Table 1. The composites are named as SBR/ST-x, where x means x phr of silica-s-TP. Then, the prepared compounds were hot-pressed at 160 °C for the optimum curing time. Then, the samples were press-cured to a 1 mm thick sheet at 160 °C and cut into the shape of dumbbell A specimen.

### 2.4. Characterization

X-ray photoelectron spectroscopy (XPS) tests were performed on a Thermo Fisher Scientific ESCALAB 250 Xi XPS (Thermo Fisher Scientific Company, Waltham, MA, USA). Fourier transform infrared (FTIR) spectroscopy was obtained from a Bruker Vector 33 FTIR spectrometer (Bruker Technology Co., Ltd., Beijing, China) in the range of 4000 cm^−1^ to 400 cm^−1^. Thermogravimetric analysis (TGA) was carried out on NETZSCH TG209F1 (NETZSCH Group, Selb, Germany) from 30 °C to 800 °C by 10 °C/min and in N_2_ atmosphere. The UV–VIS absorption spectra of samples were obtained with Lambda 35 spectrometer (Perkin Elmer, Waltham, MA, USA), the samples were dispersed in deionized water. A Merlin scanning electron microscope (SEM) instrument (ZEISS Co. Ltd., Jena, Germany) was used to observe the morphology of the filler dispersion in the fracture surface of rubber matrix. The vulcanization characteristics of the SBR compounds were conducted on rotorless rheometer UR-2030 (U-CAN DYNATEX INC., Taipei, Taiwan). Tear and tensile tests were performed on a U-CAN UT-2060 instrument (U-CAN DYNATEX INC., Taipei, Taiwan) according to standard ISO 37-2005. The crosslink density of the samples was measured by the equilibrium swelling method as reported previously [25]. Dynamic mechanical analyzer (DMA) was measured with a TA Q800 dynamic mechanical analyzer (TA Instruments, Shanghai, China) from −80 °C to 80 °C by 2 °C/min. For UV aging test, the SBR composites were placed in an UV aging testing machine (Dongguan Zhenglan Precision Instruments Co., Ltd., Dongguan, China) for 1, 2, and 3 d at 50 °C. The UV radiation intensity was 0.83 W/m^2^.

The glass transition of neat SBR and SBR/silica-s-TP composites were detected by NETZSCH DSC 204 F (NETZSCH Group, Selb, Germany). Firstly, the composites were isothermal at −80 °C for 5 min, and followed by heating to 30 °C at a rate of 10 °C/min under a N_2_ flow. Then, the experimental parameters were assigned to the heat capacity step ΔC_pn_ and the weight fraction of the immobilized polymer layer χ_im_ [26,27,28]. ΔC_pn_ and χ_im_ were calculated as follows:

(1) $$\Delta C_{\mathrm{pn}}=\Delta C_{p}/\left(1-w\right)$$

(2) $$\chi_{\mathrm{im}}=(\Delta C_{p0}-\Delta C_{\mathrm{pn}})/\Delta C_{p0}$$

where, ΔC_p0_ and ΔC_p_ were the heat capacity jump at the glass transition region of unfilled and filled polymer composites [29,30,31]. w was the weight fraction of filler in rubber compounds.

## 3. Results and Discussion

### 3.1. Characterization of Silica-s-TP

**Figure 2:**
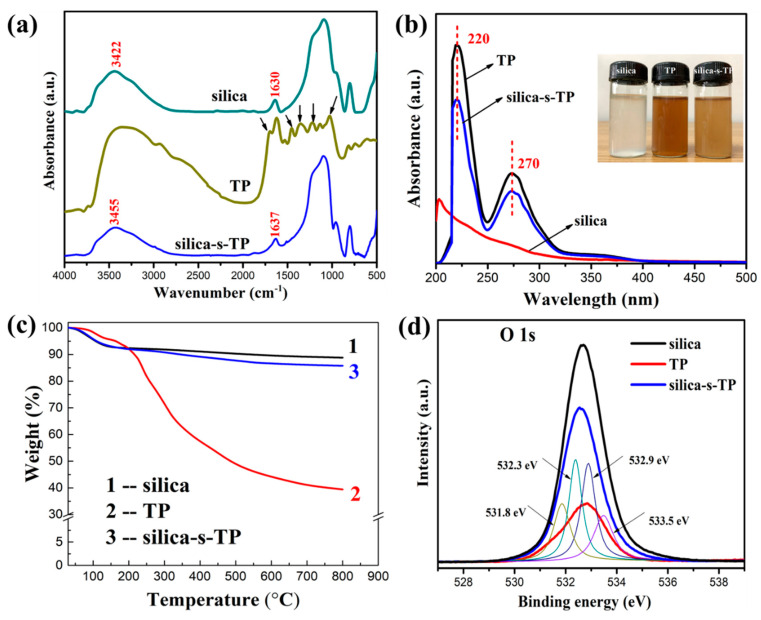
(**a**) FTIR spectra, (**b**) UV–VIS absorption spectra, (**c**) TGA curves, and (**d**) XPS O 1s spectra of pristine silica, TP and silica-s-TP (the peak fittings of silica-s-TP in the thin curves).

Figure 2a illustrated the FTIR spectra of pristine silica, TP and silica-s-TP, respectively. The spectrum for silica in the presence of characteristic peaks at 3440 cm^−1^ and 1630 cm^−1^ are, respectively, owned to the hydroxyl group stretching for silanol hydroxyls and the hydroxyl group bending of absorbed water on silica surface [27]. As shown in the infrared spectrum of TP, the typical peaks at 3340 cm^−1^ and 1348 cm^−1^ are attributed to the free or intramolecular hydrogen-bonded stretching and bending, respectively. In addition, the peaks at 1698 cm^−1^, 1621 cm^−1^, and 1448 cm^−1^ are attributed to C=O stretching, C=C vibration on the ring and C−H bending, respectively. Meanwhile, the peaks at 1144 cm^−1^ and 1034 cm^−1^ are all attributed to the C−O−C stretching [32]. Comparing the silica-s-TP with the pure TP, the infrared spectrum of silica-s-TP shows a typical similar spectrum with silica. The characteristic peaks of TP are invisible in the spectrum of silica-s-TP due to slight amount of TP grafted onto the silica surface. More sensitive detection on silica-s-TP surface can illustrate the surface structure of silica-s-TP.

The conversion of TP into silica-s-TP is manifested by UV–VIS spectroscopy in Figure 2b. The samples of silica, TP and silica-s-TP are dispersed in deionized water. The spectrum of silica shows no obvious absorption in the typical ultraviolet absorption range. The absorption peak of TP at 220 and 270 nm was assigned to π-π^*^ and n-π^*^ transition of the conjugated structure in benzene from TP [19]. The silica-s-TP appeared similar absorption to TP at 220 and 270 nm as well. This clearly illustrates that TP has successfully grafted on silica surface with the hydroxyl groups.

Thermogravimetric analysis was applied to estimate the content of TP supported onto the surface of silica particles and the curves of silica, TP and silica-s-TP were exhibited in Figure 2c. The thermogravimetric curve of silica-s-TP can be divided into two stages during the temperature range from 30 to 800 °C. The first stage below 150 °C was attributed to the dehydration of adsorbed water and the removal of silanol groups on the surface of silica. Then the stage above 200 °C was ascribed to thermal decomposition of grafted TP molecules. The loading efficiency calculated with the Equation (3) [33]:

(3) $${\mathrm{LE}=(W}_{C}-W_{A\text{-}C})/{(W}_{C}-W_{A})$$

LE: Loading Efficiency;
W_C_: the weight of silica at 800 °C;
W_A-C_: the weight of TP molecule loaded on silica (silica-s-TP) at 800 °C;
W_A_: the weight of TP molecule at 800 °C.

And the calculated value of immobilized TP on nano-silica surface was approximately 3.4 wt %.

XPS measurement on the surface characterization of the samples is more sensitive [34]. The O 1s spectra of silica, TP and silica-s-TP, and peak fittings of silica-s-TP (thin curves) are shown in Figure 2d in thin curves. As Figure 2d shown, the main peak of O 1s in silica at 532.6 eV is assigned to Si−O−H. Compared with silica, the binding energy of O 1s for silica-s-TP is decreased attribute to the chemical reaction between Si-OH and TP. While the peak could be divided into four types of oxygen of C−O−H, Si−O−C, C−O−C, and −C=O at the binding energies of 531.8, 532.3, 532.9, and 533.5 eV, respectively. This is consistent with the chemical reaction between the Si-OH group and TP to generated oxygen atoms with different binding energies [35]. Therefore, the XPS results further demonstrate that the successful bonding of TP on the silica surface.

### 3.2. Morphology of SBR Composites

**Figure 3:**
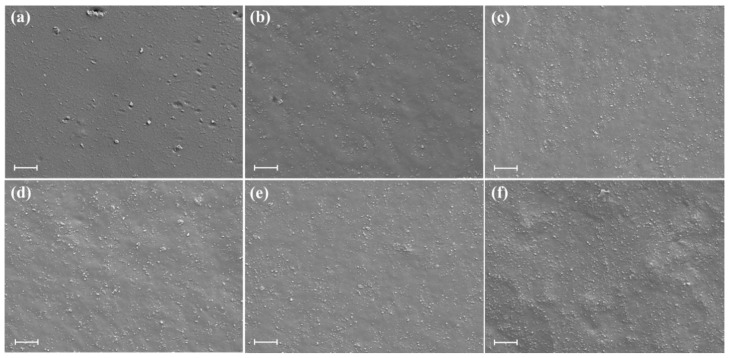
SEM images of SBR composites: (**a**) neat SBR, (**b**) SBR/ST-10, (**c**) SBR/ST-20, (**d**) SBR/ST-30, (**e**) SBR/ST-40, and (**f**) SBR/ST-50, the scale bar is 2 µm.

Figure 3 shows the SEM photos of neat SBR and SBR/silica-s-TP composites with the gradually increase amount of silica-s-TP. From the brittle cross-section of SBR as shown in Figure 3a, the cross-section of matrix expresses continuous and nearly smooth except a few ZnO and other rubber additives agglomerates. As a novel kind of functional rubber filler, the dispersion properties of silica-s-TP in SBR matrix are enhanced with the increasing filler content as displayed in Figure 3b–f. Compared with neat SBR matrix, the brittle cross-section of SBR/silica-s-TP composites becomes rough, and this morphology resembles other related reports regarding rubber/silica composites [36,37,38]. Obviously, there are no obvious aggregates exhibited in the SBR/silica-s-TP composites. Even with the increase of the added amount of silica-s-TP, the dispersion of biomass anti-aging filler in the rubber matrix is fairly uniform and without obvious aggregate formation. Furthermore, the grafted TP molecules cannot only reduce the content of hydroxyl groups on silica surface, but also act as spacers to prevent the silica particles aggregating in the rubber matrix [39,40,41].

### 3.3. Interfacial Interaction between Biomass Anti-Aging Filler and Rubber

**Figure 4:**
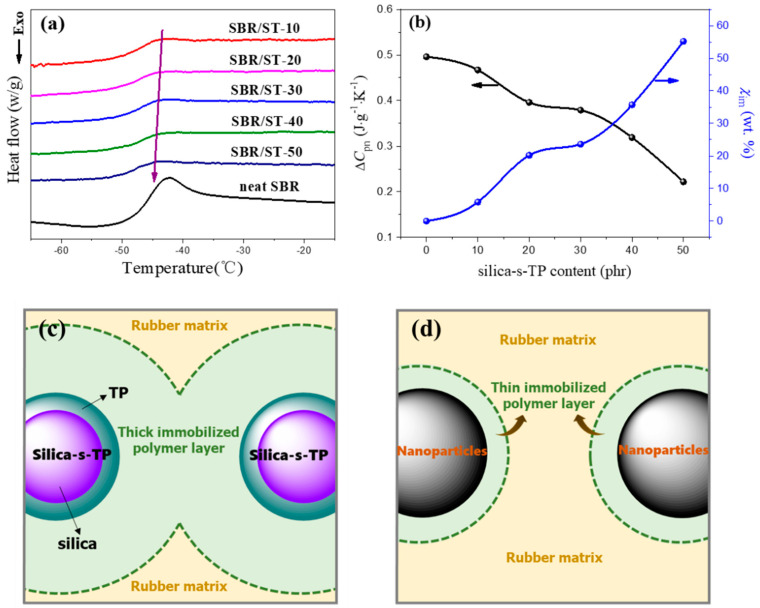
(**a**) DSC curves, (**b**) the parameters of Δ$C_{\mathrm{pn}}\;$ and $\chi_{\mathrm{im}}\;$ of neat SBR and SBR/silica-s-TP composites, (**c**) the schematic representation of the immobilized polymer layer on silica-s-TP surface in rubber matrix and (**d**) the thin immobilized polymer layer on unmodified nanoparticles surface in SBR matrix.

Rubber molecular chains have a unique long-chain sharp which are sensitive to the local condition [42]. Hence, the variation morphology of rubber chain during the glass transition process can be illustrate by the heat capacity of SBR composite [43]. The DSC curves of neat SBR and SBR/silica-s-TP composites in the glass transition region are shown in Figure 4a. From the values of Δ$C_{\mathrm{pn}}$ shown in Figure 4b are in regular sequence of neat SBR > SBR/ST-10 > SBR/ST-20 > SBR/ST-30 > SBR/ST-40 > SBR/ST-50, suggesting that the rubber chain is restricted between filler interstice with the increasing content of silica-s-TP, which giving a considerable influence on the glass transition. The variational $\chi_{\mathrm{im}}$ of the filled SBR composites are shown in Figure 4b [44] also illustrate that the motion ability of polymer chain is decreased with the increasing amount of anti-aging silica-s-TP. Meanwhile, the schematic representation of immobilized polymer layer on silica-s-TP or unmodified nanoparticles surface in SBR have exhibited in Figure 4c,d. The thicker immobilized polymer layer on silica-s-TP surface makes the combination of biomass filler and rubber matrix more tightly. Moreover, due to the filler particles surface modified by TP, the improved interfacial interaction between anti-aging filler and rubber matrix brings a mass of rubber molecular chains entangled onto silica-s-TP surface, which makes rubber chain segment tough to relax during the glass transition region and bring to the lower heat capacity. The plentiful immobilized polymer layer as a type of surface modifier to generate an intense filler-rubber interfacial interaction and to enhance the physical properties of SBR/silica-s-TP composites [34].

### 3.4. Aging Resistance of SBR Composites Filled with Anti-Aging Filler

**Figure 5:**
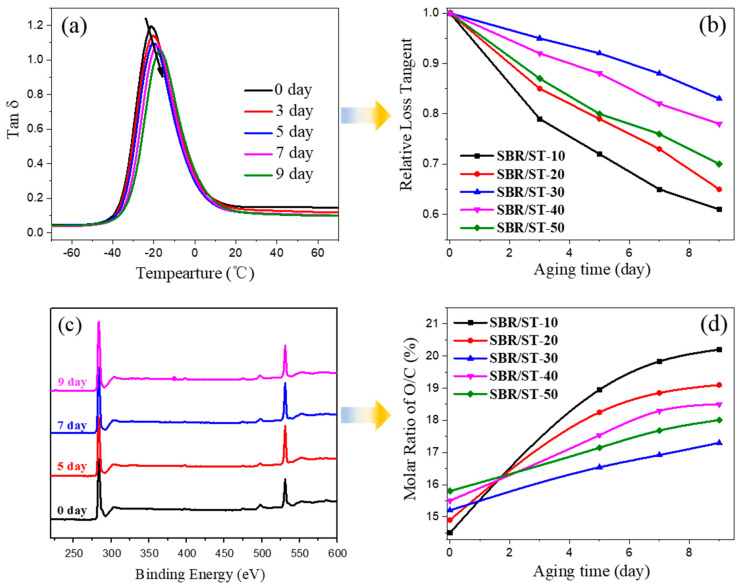
(**a**) DMA curves of tan δ vs. temperature of SBR/ST-30 with different thermo-oxidative aging time, (**b**) Relative tan δ peak value for SBR/silica-s-TP composites vs. aging time, (**c**) XPS spectrum of SBR/ST-30 during aging at 100 °C for various times, and (**d**) molar ratio of O/C of SBR/silica-s-TP composites vs. aging time.

Retarding aging is crucial for practical applications of all polymers, especially rubber materials with unsaturated carbon-carbon double bonds. DMA tests was used to reveal the affection of thermo-oxidative aging of SBR composites on the chain movement [45]. The DMA curves of SBR/ST-30 with different thermo-oxidative aging time are shown in Figure 5a, and the peak value of loss tangent (tan δ) vs. different aging times for SBR/silica-s-TP composites are exhibited in Figure 5b. The peak values of SBR/silica-s-TP composites had a moderate decline with the increasing of aging time (Figure 5a), and adding 30 phr of silica-s-TP could achieve the minimal decline (Figure 5b) due to the plentiful phenolic hydroxyl groups stem from tea polyphenols supported on silica surface which can capture the free radicals generated from the break of rubber molecular chain during thermo-oxidative aging, and further to restrict the excessive cross-linking. However, with the silica-s-TP content increasing to 40 or 50 phr, the peak values of the samples decline sharply that is probably due to the increased amount of rigid filler content can greatly limit the shift of the rubber chains. Hence, an appropriate amount of silica-s-TP can provide long-term protection through inhibiting the free radicals generated during thermo-oxidative aging [45].

To evaluate the effect of the biomass anti-aging nanofiller silica-s-TP dispersing in the rubber matrix on the long-time anti-aging, XPS tests were used to observe the oxygen diffusion process of SBR/silica-s-TP composites with different filler content after cumulative aging time. The XPS spectrum of SBR/ST-30 during aging at 100 °C in zero, five, seven, and nine days, respectively, is shown in Figure 5c. The corresponding molar ratio of O/C for the SBR/silica-s-TP composites with various aging time is shown in Figure 5d. Consistent with the above dynamic mechanical analysis results, the increase of O/C ratio for SBR/ST-30 shows the lowest, revealing the long-term antioxidative protection for SBR matrix is achieved by the adding 30 phr biomass anti-aging filler.

**Figure 6:**
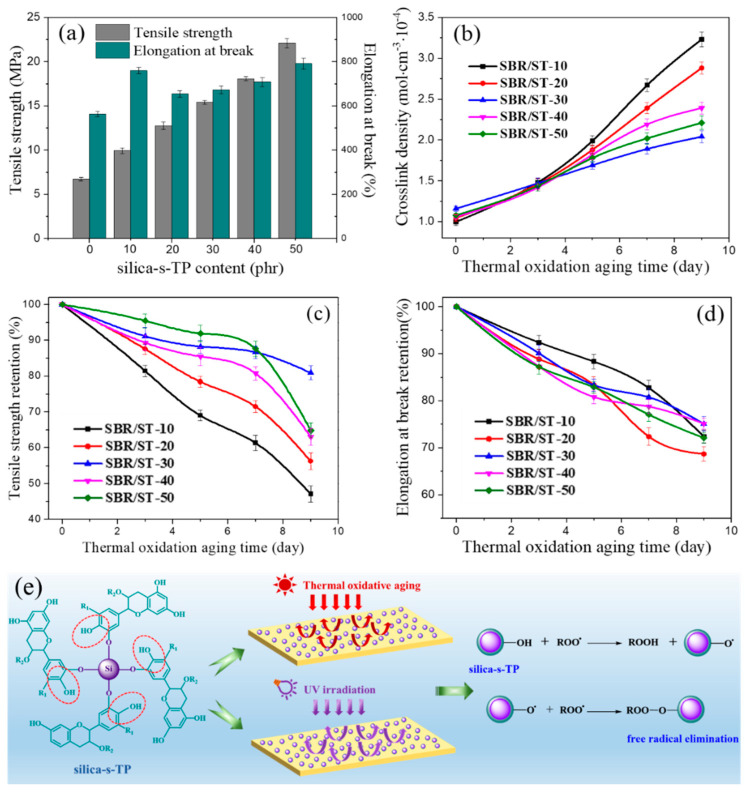
(**a**) The mechanical properties of SBR/silica-s-TP composites; (**b**) the crosslink density, (**c**,**d**) the retention of mechanical properties of SBR/silica-s-TP composites during thermo-oxidative aging at 100 °C, (**e**) the schematic representation of the mechanism of silica-s-TP in rubber matrix to prevent thermal oxidative aging and UV irradiation.

As a novel type of anti-aging filler, the anti-aging and reinforcing properties of the direct incorporation of silica-s-TP in the rubber matrix are extremely important factors to evaluate its performance. Hence, the anti-aging properties of SBR/silica-s-TP composites were assessed through comparing the variation of mechanical properties during thermal-oxidative aging at 100 °C for gradually increasing days is shown in Figure 6. Before thermal-oxidative aging, the tensile strength of rubber composites was gradually enhanced with the anti-aging biomass filler amount increasing (Figure 6a). Compared to the unfilled SBR, the tensile strength of SBR/ST-50 has almost a four-fold increasing, and extremely likely ascribe to the enhanced rubber-filler interfacial interaction and the excellent reinforcing performance of silica-s-TP as biomass filler in the rubber matrix. After thermal-oxidative aging, the recombination of the fractured short rubber chain makes the gradual increase in the crosslink density of all SBR composites (Figure 6b) [46]. For the slowest increase of SBR/ST-30 crosslink density, it can be concluded that 30 phr of silica-s-TP anti-aging effect is excellent in the rubber matrix. Furthermore, the retention of mechanical properties of SBR/silica-s-TP composites has shown the direct assessment of the aging process: the oxidation resistance of all SBR samples decreases during the extension of thermal-oxidative aging time and leading to a significant decrease in the tensile strength and elongation at break as demonstrated in Figure 6c,d. In particular, the rate of decrease of SBR/ST-30 composite is the slowest, and the tensile strength retention rate can remain above 80% and the relative elongation at break can be kept above 75% after nine days of aging. This indicates that the incorporation of 30 phr silica-s-TP into rubber matrix offers a long-term anti-aging activity that retards the aging process. In addition, the mechanism of silica-s-TP in rubber matrix to prevent thermal oxidative aging and UV irradiation has exhibited in Figure 6e. The structure of the biomass anti-aging filler is probably similar to the hindered phenolic antioxidant. When the SBR/silica-s-TP sample was exposed to thermal oxidation or UV irradiation, the hindered phenolic hydroxyl group on the surface of silica-s-TP is extremely unstable and easy to lose electrons, and the per-oxyradical formed by the breakage of rubber molecular chain can be quickly captured which leads to free radical elimination. Therefore, the biomass antioxidant of silica-s-TP cannot only effectively improve the anti-aging property of rubber, but also strengthen the physical mechanical property of the rubber matrix as a kind of biomass nanofiller.

**Figure 7:**
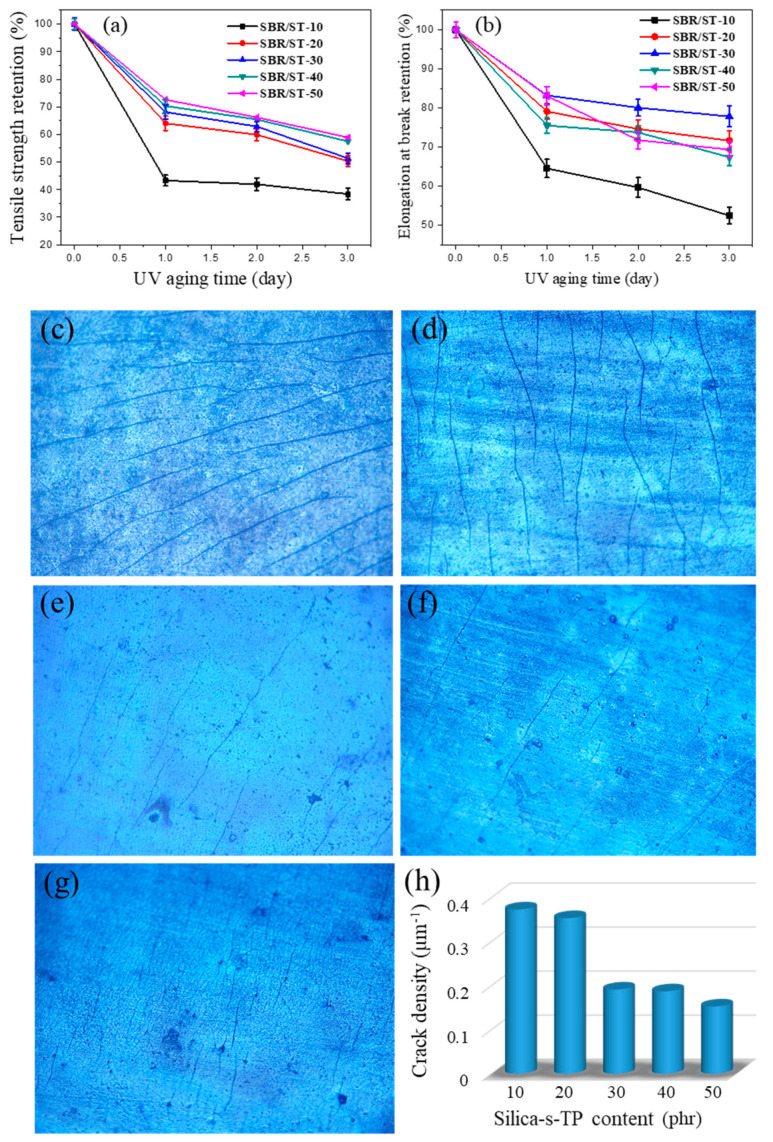
(**a**,**b**) The retention of mechanical properties of SBR/silica-s-TP composites before and after UV aging at 50 °C; (**c**–**g**) the optical photomicrographs (50-fold) of UV exposed (3 d) SBR composites containing different silica-s-TP content: (**c**) 10 phr, (**d**) 20 phr, (**e**) 30 phr, (**f**) 40 phr and (**g**) 50 phr; (**h**) the tendency chart of crack density vs. silica-s-TP content.

Figure 7a,b exhibit the retentions of tensile strength and elongation at break for SBR/silica-s-TP sheets after UV aging for one, two, and three days. Obviously, ultraviolet had a critical impact on the mechanical performance of all SBR/silica-s-TP samples. The retentions of tensile strength and elongation at break of SBR/silica-s-TP composites decreased rapidly with the UV aging time increasing, due to the fragmentation of rubber macromolecular chains. However, with the increasing content of anti-aging biomass filler, the SBR composites incorporation of silica-s-TP exhibited preferable aging resistance efficiency during the long-term ultraviolet exposure. Unsurprisingly, the tensile strength and elongation at break of SBR/ST-30 composite both remain at 55% and 77%, proving the excellent UV anti-aging efficiency of silica-s-TP. The optical photographs of SBR composites surface after three days of UV exposed are displayed in Figure 7c–g. For SBR incorporated with silica-s-TP content over 20 phr composites, the cracks are shallow and discontinuous. To the contrary, deep and continuous cracks are detected on the composites surface which incorporated with lower content of silica-s-TP. It is likely due to the higher content of silica-s-TP brings to abundance of TP in these composites to prevent the growth of cracks along the polymers. As the Figure 7h shown, the crack density of each samples shows a sharp decline tendency after adding the filler content exceed 20 phr per 100 phr rubber. The growing cracks in the reaction process will terminate due to encountering the inert particles, and the cracks only possible to further expand by breaking or skipping the inert particles [46]. Hence, silica-immobilized TP in moderation ensured a more stable and homogeneous distribution of biomass anti-aging filler in the SBR matrix, giving rise to the outstanding anti-aging property than the insufficient filling samples.

## 4. Conclusions

In summary, a novel hybrid biomass anti-aging nanofiller for improving the thermal-oxidative stability and UV aging resistance of SBR without adding other traditional small molecule antioxidant has been reported, on account of green tea polyphenols immobilized on silica surface. Functionalization of silica surface with TP demonstrated the desirable property of exhibiting improved thermal-oxidative stability especially adding 30 phr silica-s-TP into SBR matrix. Furthermore, with the increasing content of silica-s-TP, the property of UV aging resistance has been increased gradually. Unlike the traditional low molecule antioxidant, silica-s-TP has not only shown the outstanding filler dispersion, rubber-filler interfacial interaction, but also exhibited the improving stability and volatility. The results also offer inspirations of applying the biomass anti-aging material in green tire, eco-friendly rubber additives, and functional nanofiller areas.

## Figures and Tables

**Table 1:** Composition of SBR/silica-s-TP composites (phr).

	SBR	Silica-s-TP	ZnO	CBS	SA	S
Neat SBR	100	0	4	2	2	1.6
SBR/ST-10	100	10	4	2	2	1.6
SBR/ST-20	100	20	4	2	2	1.6
SBR/ST-30	100	30	4	2	2	1.6
SBR/ST-40	100	40	4	2	2	1.6
SBR/ST-50	100	50	4	2	2	1.6

## References

[1] Garbarczyk M., Kuhn W., Klinowski J., Jurga S. (2002). Characterization of aged nitrile rubber elastomers by NMR spectroscopy and microimaging. Polymer.

[2] Mitani M.M., Keller A.A., Bunton C.A., Rinker R.G., Sandall O.C. (2002). Kinetics and products of reactions of MTBE with ozone and ozone/hydrogen peroxide in water. J. Hazard. Mater..

[3] Mathew N.M., De S.K. (1983). Thermo-oxidative ageing and its effect on the network structure and fracture mode of natural rubber vulcanizates. Polymer.

[4] Munteanu S.B., Brebu M., Vasile C. (2005). Thermal and thermo-oxidative behavior of butadiene-styrene copolymers with different architectures. Polym. Degrad. Stab..

[5] Chen L.J., Guo X.H., Luo Y.F., Jia Z.X., Chen Y.J., Jia D.M. (2018). Inorganic and organic hybrid nanoparticles as multifunctional crosslinkers for rubber vulcanization with high-filler rubber interaction. Polymers.

[6] Nagle D.J., Celina M., Rintoul L., Fredericks P.M. (2007). Infrared microspectroscopic study of the thermo-oxidative degradation of hydroxy-terminated polybutadiene/isophorone diisocyanate polyurethane rubber. Polym. Degrad. Stab..

[7] Abdelwahab N.A., El-Nashar D.E., El-Ghaffar M.A. (2011). Polyfuran, polythiophene and their blend as novel antioxidants for styrene-butadiene rubber vulcanizates. Mater. Des..

[8] Luo K.Q., You G.H., Zhao X.Y., Lu L., Wang W.C., Wu S.Z. (2019). Synergistic effects of antioxidant and silica on enhancing thermo-oxidative resistance of natural rubber: Insights from experiments and molecular simulations. Mater. Des..

[9] Yamano T., Shimizu M. (2010). Skin sensitization potency and cross-reactivity of p-phenylenediamine and its derivatives evaluated by non-radioactive murine local lymph node assay and guinea-pig maximization test. Contact Dermat..

[10] Lu L., Luo K.Q., Yang W., Zhang S.D., Wang W.C., Xu H.Y., Wu S.Z. (2020). Insight into the anti-aging mechanisms of naturalphenolic antioxidants in natural rubber compositesusing a screening strategy based on molecularsimulation. RSC Adv..

[11] Zhang L., Li H.Q., Lai X.J., Wu W.J., Zeng X.R. (2018). Hindered phenol functionalized graphene oxide for natural rubber. Mater. Lett..

[12] Zhou J.J., Wei L.Y., Wei H.T., Zheng J., Huang G.S. (2017). The synthesis of graphene-based antioxidants to promote anti-thermal properties of styrene-butadiene rubber. RSC Adv..

[13] Nie J.D., Huang X.H., Xu C.H., Ding J.P., Chen Y.K. (2020). Antioxidant effects on curing/processing and thermo-oxidative aging of filled nitrile rubber. Mater. Chem. Phys..

[14] Bansal S., Syan N., Mathur P., Choudhary S. (2012). Pharmacological profile of green tea and its polyphenols: A review. Med. Chem. Res..

[15] Pasrija D., Ezhilarasi P.N., Indrani D., Anandharamakrishnan C. (2015). Microencapsulation of green tea polyphenols and its effect on incorporated bread quality. LWT Food. Sci. Technol..

[16] Chen W.R., Zhang Z.Z., Shen Y.W., Duan X.W., Jiang Y.M. (2014). Effect of tea polyphenols on lipid peroxidation and antioxidant activity of litchi (Litchi chinensis Sonn.) fruit during cold storage. Molecules.

[17] Heim K.E., Tagliaferro A.R., Bobilya D.J. (2002). Flavonoid antioxidants: Chemistry, metabolism and structure-activity relationships. J. Nutr. Biochem..

[18] Wu W.B., Chiang H.S., Fang J.Y., Chen S.K., Huang C.C., Hung C.F. (2006). (+)-Catechin prevents ultraviolet B-induced human keratinocyte death via inhibition of JNK phosphorylation. Life Sci..

[19] Liao R.J., Tang Z.H., Lei Y.D., Guo B.C. (2011). Polyphenol-reduced graphene oxide: Mechanism and derivatization. J. Phys. Chem. C.

[20] Yan Q., Wang M.Y., Wu Y.H., Shen Q. (2016). Tea polyphenol as environmentally friendly dopant and thermal stabilizer for polyaniline. Mater. Lett..

[21] Yang Z.J., Xu Z.C., Zhang L.Q., Guo B.C. (2018). Dispersion of graphene in chlorosulfonated polyethylene by slurry compounding. Compos. Sci. Technol..

[22] Liao R.J., Tang Z.H., Lin T.F., Guo B.C. (2013). Scalable and versatile graphene functionalized with the Mannich condensate. ACS Appl. Mater. Interfaces.

[23] Pan Q.W., Wang B.B., Chen Z.H., Zhao J.Q. (2013). Reinforcement and antioxidation effects of antioxidant functionalized silica in styrene-butadiene rubber. Mater. Des..

[24] Zhong B.C., Shi Q.F., Jia Z.X., Luo Y.F., Chen Y.J., Jia D.M. (2014). Preparation of silica-supported 2-mercaptobenzimidazole and its antioxidative behavior in styrene-butadiene rubber. Polym. Degrad. Stab..

[25] Zhong B.C., Jia Z.X., Hu D.C., Luo Y.F., Jia D.M. (2015). Reinforcement and reinforcing mechanism of styrene-butadiene rubber by antioxidant-modified silica. Compos. Part A Appl. Sci. Manuf..

[26] Guo L.L., Lei H.X., Zheng J., Huang G.S. (2013). Synthesis of nanosilica-based immobile antioxidant and its antioxidative efficiency in SBR composites. Polym. Compos..

[27] Chen L.J., Guo X.H., Jia Z.X., Tang Y.H., Wu L.H., Luo Y.F., Jia D.M. (2018). High reactive sulphide chemically supported on silica surface to prepare functional nanoparticle. Appl. Surf. Sci..

[28] Gao X.W., Hu G.J., Qian Z.Z., Ding Y.F., Zhang S.M., Wang D.J., Yang M.S. (2007). Immobilization of antioxidant on nanosilica and the antioxidative behavior in low density polyethylene. Polymer.

[29] Sun Y.K., He J.W., Zhong B.C., Zhu L.X., Liu F. (2019). A synthesized multifunctional rubber additive and its improvements on the curing and antioxidative properties of styrene-butadiene rubber/silica composites. Polym. Degrad. Stab..

[30] Rooj S., Das A., Stöckelhuber K.W., Wang D.Y. (2013). Understanding the reinforcing behavior of expanded clay particles in natural rubber compounds. Soft Matter.

[31] Li Y.Q., Ishida H. (2005). A Study of Morphology and Intercalation Kinetics of Polystyrene-Organoclay Nanocomposites. Macromolecules.

[32] Feng L., Li J.F., Ye J.R., Song W., Jia J., Shen Q. (2014). Enhancing the mechanical and thermal properties of polyacrylonitrile through blending with tea polyphenol. J. Appl. Polym. Sci..

[33] Chen L.J., Jia Z.X., Tang Y.H., Wu L.H., Luo Y.F., Jia D.M. (2017). Novel functional silica nanoparticles for rubber vulcanization and reinforcement. Compos. Sci. Technol..

[34] Chen L.J., Jia Z.X., Guo X.H., Zhong B.C., Chen Y.J., Luo Y.F., Jia D.M. (2018). Functionalized HNTs nanocluster vulcanized natural rubber with high filler-rubber interaction. Chem. Eng. J..

[35] Sileika T.S., Barrett D.G., Zhang R., Lau K.H.A., Messersmith P.B. (2013). Colorless multifunctional coatings inspired by polyphenols found in tea, chocolate, and wine. Angew. Chem. Int. Ed..

[36] Peng Z.C., Li Q., Li H.Y., Hu Y.L. (2017). Polyethylene-modified nano silica and its fine dispersion in polyethylene. Ind. Eng. Chem. Res..

[37] Yan F.H., Zhang X.B., Liu F., Li X.H., Zhang Z.J. (2015). Adjusting the properties of silicone rubber filled with nanosilica by changing the surface organic groups of nanosilica. Compos. Part B Eng..

[38] Rahman I.A., Jafarzadeh M., Sipaut C.S. (2009). Synthesis of organo-functionalized nanosilica via a co-condensation modification using γ-aminopropyltriethoxysilane (APTES). Ceram. Int..

[39] Li Y., Han B.Y., Wen S.P., Lu Y.L., Yang H.B., Zhang L.Q., Liu L. (2014). Effect of the temperature on surface modification of silica and properties of modified silica filled rubber composites. Compos. Part A Appl. Sci. Manuf..

[40] Li Y., Han B.Y., Liu L., Zhang F.Z., Zhang L.Q., Wen S.P., Lu Y.L., Yang H.B., Shen J. (2013). Surface modification of silica by two-step method and properties of solution styrene butadiene rubber (SSBR) nanocomposites filled with modified silica. Compos. Sci. Technol..

[41] Liu X., Zhao S.H., Zhang X.Y., Li X.L., Bai Y. (2014). Preparation, structure, and properties of solution-polymerized styrene-butadiene rubber with functionalized end-groups and its silica-filled composites. Polymer.

[42] Zhang X.G., Loo L.S. (2009). Study of glass transition and reinforcement mechanism in polymer/layered silicate nanocomposites. Macromolecules.

[43] Zou Y.K., Sun Y.K., He J.W., Tang Z.H., Zhu L.X., Luo Y.F., Liu F. (2016). Enhancing mechanical properties of styrene–butadiene rubber/silica nanocomposites by in situ interfacial modification with a novel rare-earth complex. Compos. Part A Appl. Sci. Manuf..

[44] Begum P.M.S. (2011). Use of antioxidant-modified precipitated silica in natural rubber. Prog. Rubber. Plast. Recycl. Technol..

[45] Wu S.W., Qiu M., Guo B.C., Zhang L.Q. (2017). Nanodot-loaded clay nanotubes as green and sustained radical scavengers for elastomer. ACS Sustain. Chem. Eng..

[46] Vinod V.S., Varghese S., Kuriakose B. (2002). Degradation behaviour of natural rubber-aluminium powder composites: Effect of heat, ozone and high energy radiation. Polym. Degrad. Stab..

